# Incorporation of Poly(propylene succinate-*co*-glycerol succinate) (PPSG) as a Renewable Additive in Electrospun PCL Fibers with Bioactive Glass Particles for Soft Tissue Engineering

**DOI:** 10.1021/acsabm.5c00176

**Published:** 2025-06-03

**Authors:** Clara Dourado Fernandes, Alina Grünewald, Zoya Hadzhieva, Bruno F. Oechsler, Claudia Sayer, Pedro H. Hermes de Araújo, Aldo R. Boccaccini

**Affiliations:** † Department of Chemical Engineering and Food Engineering, 28117 Federal University of Santa Catarina, 88040-900 Florianópolis, Santa Catarina, Brazil; ‡ Institute of Biomaterials, Department of Materials Science and Engineering, 9171 University of Erlangen-Nuremberg, Cauerstr. 6, 91058 Erlangen, Germany

**Keywords:** Sustainable electrospinning, Biodegradability, Hydrophilicity, 45S5 bioglass, Composite fibers PPSG

## Abstract

As human longevity increases, the prevalence of age-related pathologies grows, driving the need for advances in regenerative medicine. This research evaluates poly­(propylene succinate-*co*-glycerol succinate) (PPSG) as a renewable additive in electrospun polycaprolactone (PCL) mats, to develop biodegradable and biocompatible scaffolds incorporating 45S5 bioactive glass (BG) particles of size ∼4 μm. Electrospinning solutions with 20% (w/v) acetic acid were used, with PPSG proportions of 5%, 10%, and 15% by weight. Additionally, BG particles were incorporated at 5, 15, and 30 wt % to enhance bioactivity. Uniform fibers were achieved with 10% PPSG at 0.4 mL/h and 15 kV, yielding bead-free structures. PPSG increased fiber diameter and mechanical properties, with Young’s modulus (E) rising from 1.7 ± 1 MPa (pure PCL) to 8.8 ± 1.5 MPa (PCL/20PPSG). Ultimate tensile strength (Σ) improved from 0.4 MPa (PCL) to 1.5 MPa (PCL/10PPSG). BG incorporation enhanced bioactivity but reduced mechanical stability due to particle distribution. Samples containing 15% BG exhibited significantly increased NHDF cell viability, and hydrophilicity improved with PPSG (reduced from 110° to 28 ± 3°). Biodegradability testing revealed a 45% ± 5 mass loss for 10% PPSG fibers over 35 days. The PCL/PPSG/BG composite demonstrates enhanced mechanical strength, bioactivity, and cell viability, making it a promising candidate for soft tissue engineering and regenerative medicine.

## Graphical abstract

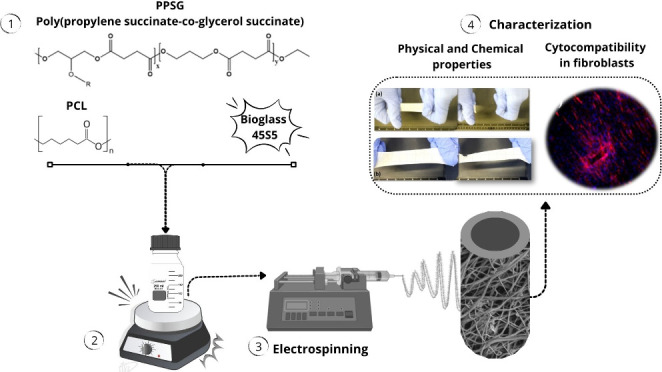

## 1 Introduction

Over the years, the global aging population has brought forth a series of medical challenges, particularly with the rise of age-related diseases such as tissue degeneration and congenital disabilities.^1^ Advances in biomedical science and technology have made it possible to replace diseased tissues with grafts and prosthetics, restoring normal biological functions. In this context, regenerative medicine has emerged as a cutting-edge field that aims to study tissue reconstitution to restore lost or compromised biological functions. A key focus within this area is tissue engineering, whose goal is to create grafts that mimic the properties of native biological tissues, providing adequate support for cellular repair and regeneration.^2,3^

Tissue engineering employs various strategies to fabricate scaffolds that promote tissue regeneration, and electrospinning has emerged as a particularly promising method. It is an efficient and versatile method capable of generating fibers usually of nanoscale diameter, from natural and synthetic polymers.^4,5^ The primary appeal of this technique lies in its ability to replicate the native cellular organism, a crucial feature for biomedical applications aimed at tissue regeneration.^6^ Moreover, the electrospinning process allows for the customization of the fibers’ physicochemical properties by adjusting parameters such as the polymer solution’s rheology, injection rate, applied voltage, and environmental conditions, including humidity and temperature.^7^ Electrospun fiber scaffolds must exhibit specific characteristics for instance, biocompatibility, controllable degradation, and adjustable mechanical properties for clinical applications.^7,8^ Biocompatibility is critical to ensure the material does not provoke adverse reactions in the human body, while biodegradability is essential because the scaffold needs to degrade in a controlled manner as the tissue regenerates.^9^ Additionally, the fibers must mimic the target tissue’s shape, microstructure, and mechanical properties, providing a conducive environment for cell attachment, migration, and proliferation.^8^ In this context, the choice of material is fundamental, and in recent years, the use of renewable polyesters represents a significant advancement for scaffold production in medical applications.

Among widely studied biodegradable polymers, poly­(glycerol sebacate) (PGS) has gained prominence for its renewable sourcing, mechanical properties akin to those of soft biological tissues, and its capacity for controlled degradation in physiological environments.^10,11^ However, the synthesis of PGS involves harsh polymerization conditions, such as high temperatures that lead to a high degree of cross-linking due to the reaction with the secondary hydroxyl of glycerol, in addition to the use of toxic catalysts, which can result in loss of glycerol and affect the final properties of the polymer.^12–14^ The slow reaction and tendency toward gelation make it challenging to produce PGS with precise control over its physicochemical properties. To overcome these limitations, the search for PGS analogs that can be synthesized under milder conditions and with greater efficiency has become a key focus in tissue engineering.^12^

In this context, a significant innovation is the enzymatic synthesis of biodegradable polyesters. This method offers a sustainable alternative to traditional chemical routes, eliminating the need for toxic solvents and reagents and allowing for more excellent modulation of the polymer structure.^15,16^ Among the new biodegradable copolymers being explored, poly­(propylene succinate-*co*-glycerol succinate) (PPSG) stands out as a promising alternative to PGS and poly­(butylene succinate) (PBS), particularly due to its processability, hydrophilicity, and tunable degradation rate.^17–19^ Unlike PGS, which requires postprocessing steps such as thermal curing or chemical cross-linking for fiber stabilization, PPSG can be directly electrospun, eliminating the need for additional chemical modifications.^20–22^ This significantly reduces processing complexity while maintaining polymer integrity, making it highly suitable for scaffold fabrication.

Furthermore, PBS, another commonly studied aliphatic polyester for biomaterial construction, exhibits high crystallinity and lower hydrophilicity, which limits its ability to support cell adhesion and bioactivity.^23,24^ In contrast, PPSG introduces structural asymmetry due to its propylene succinate units, changes the crystallinity and increasing hydrophilicity, ultimately improving the interaction between the scaffold and surrounding biological tissues (Scheme 1). PPSG also stands out due to its renewable origin and is synthesized through the enzymatic transesterification of diethyl succinate, 1,3-propanediol, and glycerol, using lipase N435.^25,26^ This method allows a high yield (94%) and results in a controlled branching of the polymer, essential to tune its mechanical properties and hydrophilicity.^27^ The presence of glycerol in the polymer structure further increases flexibility and degradation control, providing a balance between mechanical stability and bioresorption.

**Scheme 1:**
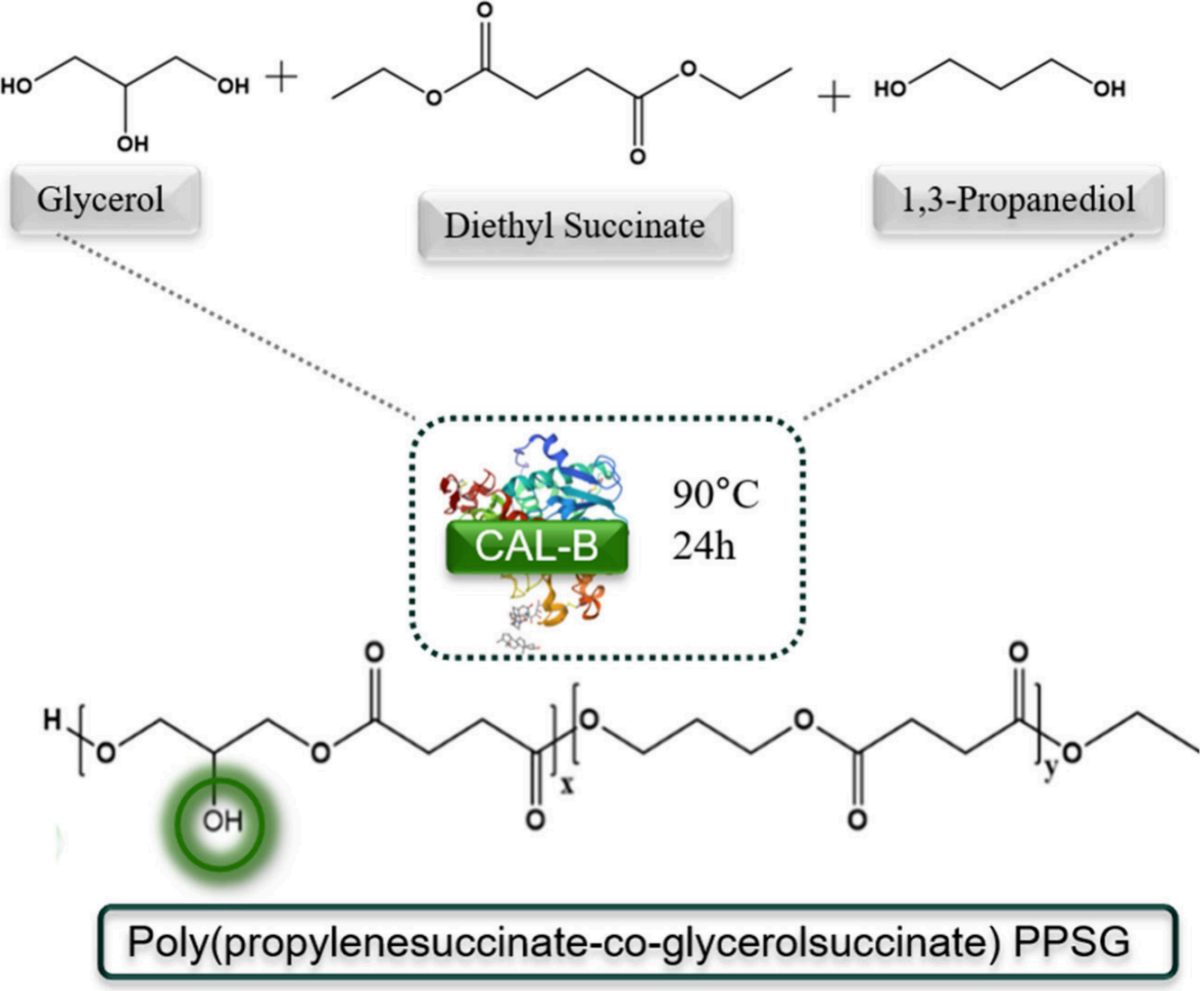
Synthesis of PPSG in Bulk Using the Immobilized Lipase N435

The branched structure of PPSG provides adjustable hydrophilicity, an essential feature for its integration into composites designed for tissue regeneration. Hydrophilicity, in turn, can facilitate cell adhesion and proliferation, as the aqueous environment is more conducive to cell-material interaction. Earlier research has demonstrated that the controlled branching of PPSG also promotes the modulation of the composite’s mechanical properties, allowing for the improvement of flexible scaffolds with appropriate characteristics for tissue regeneration, such as skin and muscle.^28,29^

Inorganic bioactive materials, such as 45S5 bioactive glass (BG), have been integrated into polyester fibers to enhance the properties of tissue regeneration scaffolds.^30^ Biologically active glasses are known for their capability to promote osteogenesis and cell adhesion as well as release therapeutic ions that stimulate cellular activity.^10^ Although 45S5 BG has been widely used in bone regeneration, its incorporation into soft tissue scaffolds also shows potential, as the ions released by the glass can mitigate the effects of acidic degradation products from biodegradable polyesters, improving the local cellular environment.^11^ Moreover, bioactive glass can increase the stiffness and mechanical strength of the matrix without compromising its flexibility thus maintaining scaffold integrity during tissue regeneration.

Electrospinning is a very common technique used to fabricate polymer fibers for tissue engineering applications.^31^ Recently, there has been interest in introducing benign solvents (less toxic solvents) in electrospinning processes.^32,33^ In light of this, the present work aims to develop a new electrospun fiber composite that combines polycaprolactone (PCL), PPSG, and bioactive glass particles using a green solvent, acetic acid. The use of acetic acid as a solvent promotes the sustainability of the production process and aligns with the increasing trend of adopting environmentally friendly methods in biomaterials science.^34–37^ The combination of PCL, PPSG, and bioactive glass should result in a composite with superior mechanical and biological properties, especially for potential targets in soft tissue repair, such as skin regeneration and for diabetic ulcers treatment.

Furthermore, this work addresses a significant gap in the literature. To our knowledge, no previous study has combined PCL, PPSG, and bioactive glass to produce electrospun fibers for soft tissue regeneration. By addressing the mechanical and biological limitations of existing tissue scaffolds, this new composite has the prospective to become a valuable biomaterial technology for biomedical applications.

## 2 Materials and Methods

### 2.1 PPSG Samples

PPSG was synthesized by enzymatic transesterification according to the protocol reported by Fernandes et al.^15^ Briefly, the monomers glycerol, 1,3-propanediol, and succinic acid (all from Sigma-Aldrich), were homogenized under N_2_ flow for 30 min. 10% (w/w) enzyme (N435) was added with an initial activity of 32.3 U.g^–1^. The reaction proceeded for 24 h at 90 °C and constant stirring under reduced pressure. PPSG has an average molecular weight (Mw) of 11 kDa, a dispersity (Đ) of ± 2, and a mean degree of polymerization (DPn) of ± 9. Based on the ^1^C NMR spectra, an enzymatic preference for the primary hydroxyls of glycerol was 94%. The presence of the unreacted tertiary hydroxyl group is used to improve hydrophilicity.^27^ The detailed protocol of enzymatic synthesis has been reported elsewhere.^15^

### 2.2 Polymer Solution Preparation and Electrospinning Process

The polymer blend solution for electrospinning was prepared using PCL (80 kDa, Sigma-Aldrich, Munich, Germany) with different concentrations of PPSG dissolved in 20% (w/v) glacial acetic acid (AA, VWR, Darmstadt, Germany). PCL refers to the blank sample without PPSG, while PCL/5PPSG, PCL/10PPSG, and PCL/20PPSG mean 5% (w/w), 10% (w/w), and 20% (w/w) PPSG, respectively. The preliminary tests were operated at the flow rates of 0.2 mL/h, 0.4 mL/h, and 0.8 mL/h. This variation was conducted to assess its effect on fiber morphology and determine the most suitable condition for generating homogeneous, bead-free fibers capable of incorporating bioactive glass nanoparticles. BG particles of 45S5 composition (in wt% 45% SiO_2_, 24,5% Na_2_O, 24,5% CaO, 6% P_2_O_5_) were used (particle size ∼ 4 μm). Samples were labeled asPCL/10PPSG/5BG, PCL/10PPSG/15BG, and PCL/10PPSG/30BG and represent 5, 15, and 30 wt % BG content relative to the polymer phase respectively. To aid in the dispersion of the glass particles, the solution was left in an ultrasonic bath for 10 min and then electrospun. All fibers were produced using an electrospinning device at (EC-CLI, IME Technologies, Netherlands) at 15 kV, relative humidity of 25 ± 0.5% and temperature of 24 ± 2 °C, with a 21G needle with the fixed collector at 11 cm distance.

### 2.3 Characterization

#### 2.3.1 Structure and Composition

The electrospun fibers were evaluated for their morphology and bioactive glass dispersion by *scanning electron microscopy (SEM)* (Auriga model from Zeiss). Before the SEM analysis, the samples were coated with gold sputtering, with the Q150T manufactured by Quorum Technologies Ltd. Then, the 100-fiber diameter was measured for each sample using the Image-J software.

*Fourier transform infrared spectroscopy (FTIR)* analysis was conducted using the IRAffinity-1S device from Shimadzu in the attenuated total reflectance (ATR) method. The device operated in the 400 to 4000 cm^–1^ wavenumber range with 4 cm^–1^ resolution, and 40 scans were used to analyze the PCL/PPSG/BG fiber mats.

*X-ray diffraction (XRD)* patterns of fibers were obtained using the MiniFlex 600 model from Rigaku, with a range of 10–80° for 2θ at a step size of 0.02° and a dwell time of 1°/min.

#### 2.3.2 Wettability

The degree of wettability of electrospun mats was obtained by measuring the angle of water drops on the fibers with 5s of stability using the DSA 2.0 instrument from Krüss GmbH. Five replicates of each composition were observed.

#### 2.3.3 Mechanical Characterization

Tensile tests were performed with dry fibers on an Instron 5960 Dual Column Table Test System. Electrospun mats (thickness 0.3 ± 0.4 mm × width 5 mm × length 20 mm) were fixed on paper frames and subjected to a tensile load of 50 N at a 1 mm.min^–1^ speed. The ultimate tensile strength (σ), Young’s modulus (*E*), and stress–strain curves were determined for all samples.

#### 2.3.4 Hydrolytic Degradation

The fiber mats were immersed in phosphate-buffered saline (PBS) (Gibco Thermo Scientific, Schwerte, Germany) for 40 days to evaluate their degradation behavior. The samples were incubated at 37 °C and 90 rpm (KS 4000 I Control, IKAWerke GmbH & Co. KG, Staufen, Germany). Every 5 days, samples were collected, washed gently with ultrapure water, and dried at room temperature. When constant weight was reached, degradation could be measured following Eq. 1:

$$Weight\;loss\;\left(\%\right)=\left(\frac{m_{1}-m_{2}}{m_{1}}\right)\cdot 100$$ 1

Where *m*_1_ is the initial mass of the fiber mat, and *m*_2_ is the mass of the dry fiber mat after degradation.

Complementary SEM and FTIR analyses were performed to investigate changes in the morphology and chemical structure of the samples.

#### 2.3.5 In Vitro Acellular Bioactivity

Electrospun mats containing of BG particles were evaluated for their acellular bioactivity by immersion tests in simulated body fluid (SBF).^38^ Triplicate samples were fixed on suitable scaffold supports and submerged in 3 mL SBF prepared according to the protocol of Kokubo et al.^39^ The samples were incubated at 37 °C under constant agitation (90 rpm in a table shake; KS 4000i control, IKA, Germany). After 1, 3, 7, and 14 days, samples were washed in ultrapure water and dried in a fume hood at room temperature for subsequent SEM (morphology), XRD, and FTIR (chemical composition changes) analysis.

#### 2.3.6 Cell Culture

Cell viability studies were performed using NHDF (normal human dermal fibroblasts; Promocell, Germany). Fiber mats were attached to PCL coronal cell inserts, which were made in-house by 3D printer (Ultimaker S5 3D printer). Fibers were previously disinfected in a UV chamber and stored in sterile 24-well plates. Cells were cultured in DMEM (Dulbecco’s Modified Eagle Medium from Gibco Thermo Scientific, Schwerte, Germany) supplemented with 1% penicillin (Thermo Scientific, Schwerte, Germany) and 10% FBS (fetal bovine serum from Corning GmbH, Wiesbaden, Germany) in 175 cm^2^ cell flasks until reaching 90% confluence within 48 h. To perform direct assays, cells were seeded at a concentration of 50,000 per 100 μL on each scaffold. After a few minutes of absorption, each well was carefully filled with 900 μL of growth medium until covering the film. The samples were kept in a sterile incubator at 37 °C with CO_2_ and humidity control, with renewal of the culture medium every 2 days. After the incubation period, the samples were stained with the WST-8 reagent kit (2-(2-methoxy-4-nitrophenyl)-3-(4-nitrophenyl)-5-(2,4-disulfophenyl)-2H tetrazolium, monosodium salt; CCK-8 kit, Sigma-Aldrich) at 5 vol%. The reaction occurred for 3 h with the samples incubated at 37 °C. Then, the viability was measured by absorbance at a wavelength of 450 nm through the spectrophotometric multiplate (FLUOstar Omega, BMG Labtech, Germany).

To investigate the morphology, the electrospun mats with grown cells were analyzed by fluorescence using an Axio Scope A1 microscope (Carl-Zeiss, Jena, Germany) following the protocol reported previously.^40^ Briefly, the samples were washed in DPBS (Dulbecco’s phosphate buffered saline from Thermo Scientific, Schwerte, Germany) and maintained under permeabilization buffer for fixation (Triton X-100, sucrose and PBS from Sigma-Aldrich). Then, the mats were stained with rhodamine-phalloidin (8 μL/mL solution) and DAPI (4′,6-diamidino-2-phenylindole, Thermo Scientific, Schwerte, Germany) (1 μL/mL solution) (Thermo Fisher Scientific).

### 2.4 Statistics

The data were presented as mean values with corresponding standard deviations. Statistical analyses were conducted using Student’s *t* test and one-way ANOVA (Origin 8.5), with significance determined for p-values less than 0.05.

## 3 Results and Discussion

### 3.1 Electrospinning of PPSG–PCL

To determine the correlation of electrospinning parameters and fiber mat structure, Figure 1 represents the interference analysis of the PPSG and BG concentration parameters and the flow rate to clarify the influence of the polymers component and BG particles on the mechanical and biological performance of the fiber mats.

**Figure 1:**
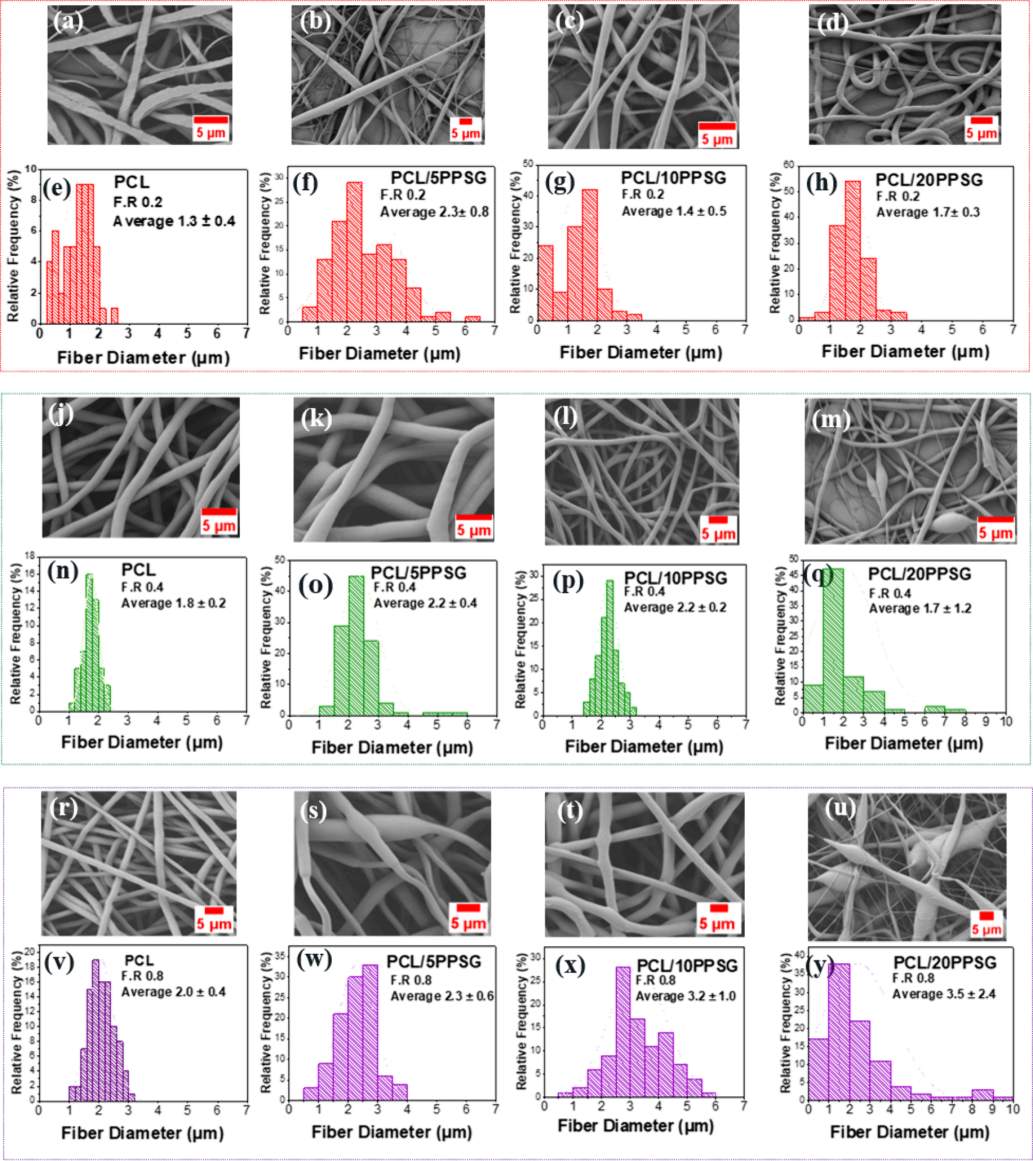
SEM micrographs of electrospun fibers and their respective average diameter distribution: (**a-h**) PCL/PPSG flow rate (F.R) 0.2 mL/h; (**j-q**) PCL/PPSG F.R 0.4 mL/h; (**r-y**) PCL/PPSG F.R 0.8 mL/h.

The analysis indicated that with the addition of PPSG, the distribution of the average fiber diameter widens. This phenomenon has been previously reported for PCL/PGS blends and is related to the modification of the polymer solution’s properties, particularly viscosity and conductivity, which influence fiber formation during electrospinning. It was observed that at higher PPSG concentrations (20% w/v), the surface tension and viscosity of the solution tend to decrease due to the lower molecular weight of PPSG compared to PCL.^41,42^ According to Fong,^41^ the alteration of these parameters causes instability in the jet caused by fluctuations in the density of electric charges. Therefore, the reduction in surface tension combined with lower viscosity and higher conductivity leads to a tendency for jet breaking, which caused the formation of beads in the PCL/20PPSG samples.

The fiber diameter distribution was more uniform when an intermedium flow rate of 0.4 mL/h was used, achieving narrow distribution diameters for PCL fibers (1.8 ± 0.2 μm), corroborating previous studies of (0.11–3.85 μm) that also used the benign solvent acetic acid.^11,40,43^ Furthermore, the average diameters of fibers composed of PPSG with 5% BG (PCL/5PPSG) of 2.2 ± 0.4 μm and 10% BG (PCL/5PPSG) with 2.2 ± 0.2 μm are in line with the literature regarding for the application of cell mats in the regeneration of fibrous structures of human intramyocardial tissue (perimysial, endomysial and epimysial fibers), ranging from 0.4 to 4 μm.^44^

The stability of the electrospinning jet and the mechanisms underlying fiber formation were strongly influenced by the composition of the polymeric solution and the processing parameters. The applied voltage of +18 kV at the needle and −2 kV at the collector, resulting in a total potential difference of 20 kV, was chosen based on previous studies that demonstrated optimal jet formation and fiber elongation under similar conditions.^34^ This voltage range ensures a balance between polymer jet stretching and solvent evaporation, minimizing droplet formation and promoting uniform fiber deposition.^34,36,45^

The addition of PPSG to the PCL matrix altered the solution properties, affecting jet stability and fiber morphology. PPSG, due to its structure and hydrophilicity, increases solution conductivity while decreasing viscosity, which are critical factors in electrospinning performance.^46,47^ At concentrations 20%, a disruption in jet stability was observed, likely caused by charge density fluctuations and insufficient viscosity to maintain a stable polymer jet. This phenomenon led to bead formation and decreased fiber uniformity, as evidenced in the SEM analysis (Figure 1). Similar trends have been reported for other polyester-based electrospun blends, where higher hydrophilic content resulted in increased jet instability.^4,8,48^

The role of solution viscosity and conductivity in jet formation is well established in the literature.^49^ While a moderate increase in viscosity enhances fiber stretching and diameter control, excessive viscosity can hinder electrospinning, leading to interrupted jets and droplet formation. The selected polymer concentrations (5%, 10%, and 20% PPSG, relative to the total polymer content) were determined based on the processability of the solution, ensuring fiber formation while avoiding excessive instability.^50^ Additionally, the fixed needle-to-collector distance of 11 cm was maintained to optimize solvent evaporation and prevent fiber fusion on the collector, as reported in previous electrospinning studies^40^

The thermal analysis (Figure S1, Table S1) corroborated the electrospinning results by demonstrating a reduction in crystallinity at higher PPSG concentrations, as evidenced by the decrease in enthalpy of fusion (ΔHf) from 3.8 J/g (PCL) to 3.1 J/g (PCL/20PPSG). The decrease in crystallinity aligns with the lower molecular weight of PPSG, reducing polymer chain entanglement likely contributed to the instability observed during fiber formation at 20% PPSG, supporting the observed bead formation and jet disruptions.

Future investigations should include a more detailed rheological characterization of the polymer solutions, evaluating viscosity, conductivity, and surface tension to better correlate solution properties with fiber formation mechanisms. Understanding these parameters could further refine the electrospinning conditions for PCL/PPSG blends and optimize scaffold morphology and performance for biomedical applications.

Preliminary tests revealed that concentrations above 20% led to significant processing challenges due to high dilution of the polymer solution. The decrease in viscosity at higher PPSG content was primarily due to the reduction in the average molecular weight of the polymer blend, as PPSG has a lower molecular weight than PCL. It is important to note that the total polymer concentration in the solution remained constant at 20% (w/v), and only the PPSG-to-PCL ratio within the polymer blend was modified. The lower viscosity at higher PPSG fractions hindered sufficient polymer chain entanglement, which is crucial for electrospinning, resulting in unstable jet formation and failure to generate continuous fibers. Furthermore, the hydrophilic nature of PPSG increases the conductivity of the solution, and at high concentrations, excessive charge repulsion disrupted fiber elongation, leading to bead formation and fiber coalescence on the collector. Given these observations, 20% PPSG was established as the upper limit for fiber formation, while 10% PPSG operated at 0.4 mL/h was identified as the optimal concentration, balancing fiber morphology, mechanical stability, and bioactivity. This selection ensures that the electrospun fibers maintain the bead-free structural integrity while incorporating sufficient PPSG to investigate the changes in fiber properties. Therefore, this condition was considered the best and was selected to be maintained in the following steps. Furthermore, the effect of 45S5 BG particles was evaluated at different concentrations (3%, 15%, and 30%) in PCL polymer solution containing 10% PPSG (samples named PCL/PPSG/3BG; PCL/PPSG/15BG; PCL/PPSG/30BG).

The analysis confirmed the presence of silica as a marker for the presence of BG particles in the fiber structures (Figure 2a–c). The addition of BG particles increased the suspension’s conductivity, which resulted in a significant change in the average diameter of the fibers (Figure 1). However, despite the smaller diameter of the fibers of samples containing BG particles (0.9 ± 0.4 μm), they are still within the application range for soft tissues engineering.^14^

**Figure 2:**
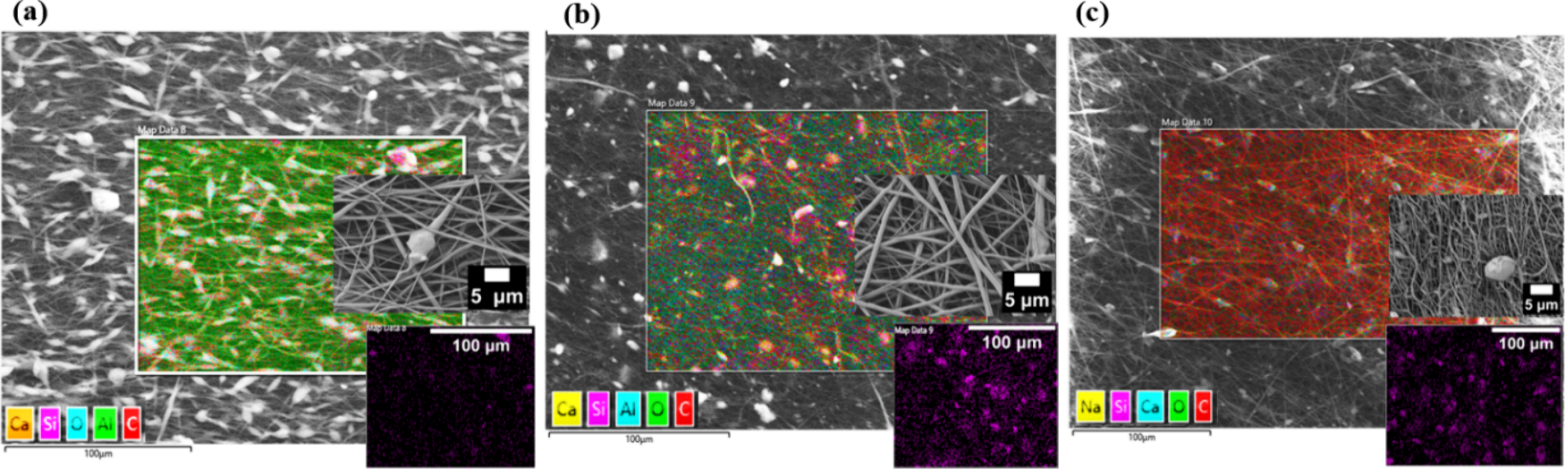
SEM/EDX showing BG particle distribution in the fiber mats: (**a**) PCL/PPSG/5BG, (**b**) PCL/PPSG/15BG, (**c**) PCL/PPSG/30BG. All compositions contain 10% PPSG.

### 3.2 Chemical Characterization

The chemical composition of the fibers was measured using FTIR spectroscopy. Figure 3 presents the spectra of fibers without any PPSG (PCL), with 10% PPSG (PCL/PPSG), and with different concentrations of BG (PCL/PPSG/BGs). At the same time, the characteristic absorption bands are detailed in Table S2. No absorption bands associated with acetic acid were detected, indicating that the solvent was eliminated.

**Figure 3:**
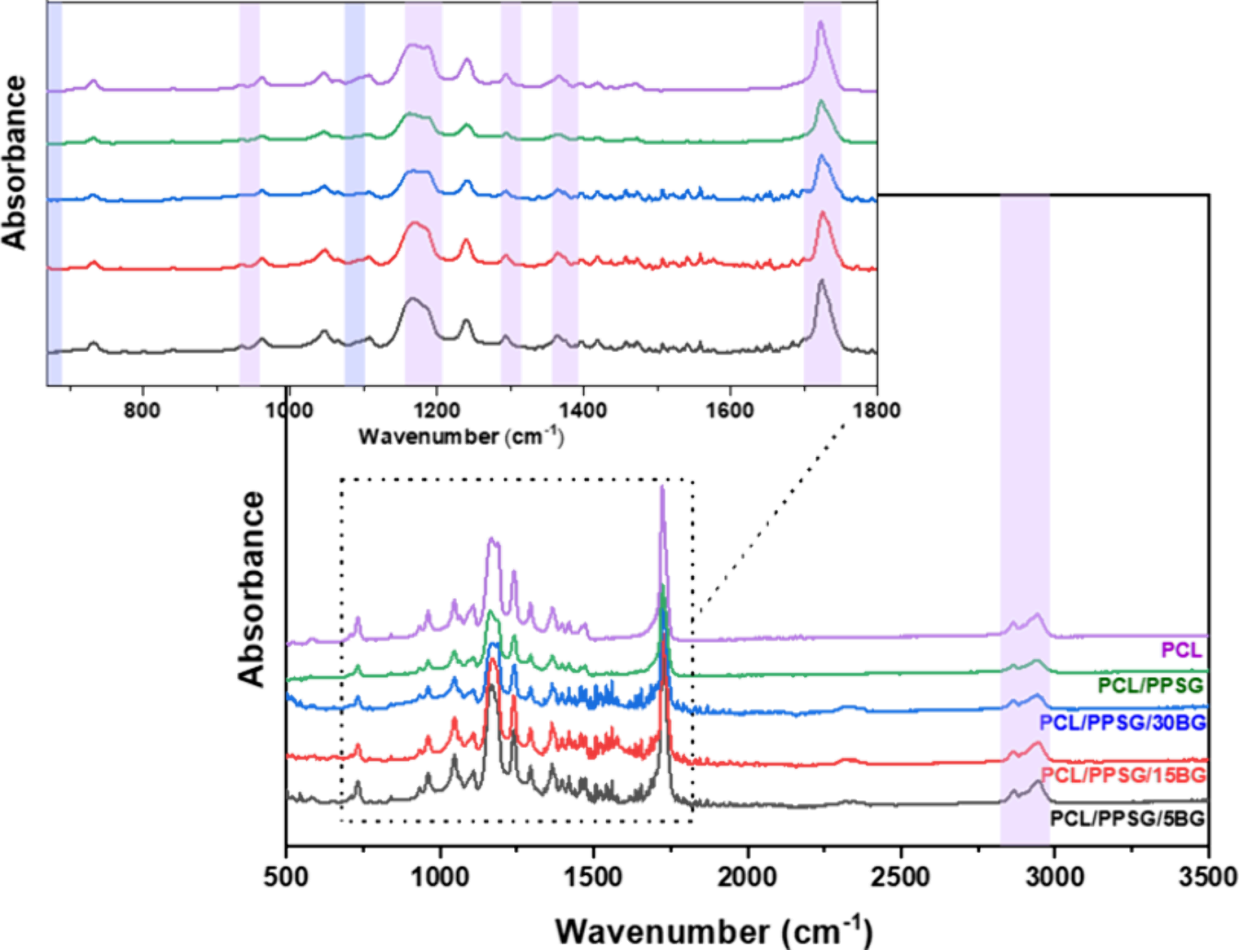
FTIR spectroscopy of fiber mats in the 400 to 3500 cm^–1^ wavenumber range. Highlights are indicated in purple and detailed in Table S2.

Analysis of the FTIR results confirms the presence of the PCL spectral features as reported in previous studies,^48,51,52^ highlighting the peaks at 2935 cm^–1^, 2864 cm^–1^, and 1365 cm^–1^, related to the CH_2_ stretching, as well as peaks at 1296 cm^–1^ and 1166 cm^–1^, allocated to asymmetric (C–O) and symmetric (C–C) stretching. At 1726 cm^–1^, the peak was referent with carbonyl stretching (C=O).^53^ Furthermore, a 1296 cm^–1^ peak was observed, attributed to stretching the C–C and C–O backbone.^54^

The overlap of the specific bands related to PPSG and BG 45S5 with the prominent peaks of PCL made their detection difficult in Figure 3. In particular, the polyester exhibits characteristic peaks around 1365 cm^–1^ and 2935 cm^–1^, corresponding to the bending absorption of CH_2_ and asymmetric deformation of the C–H bond in order.^54^ The axial stretching vibration of the C­(=O)–O bond, deemed the most distinctive diethyl succinate esters vibrational mode, is observed at approximately 1295 cm^–1^.^27^ Likewise, axial strain vibrations in the C=O functional group of diester compounds were detected at 1726 cm^–1^, consistent with the observations made for the PCL sample.

Similarly, when considering fibers containing BG45S5 particles (PCL/10PPSG/5BG, PCL/10PPSG/15BG, PCL/10PPSG/30BG), the detection of characteristic absorption bands of the silicate, such as the stretching modes Si–O–Si and Si–O bonds in the range 900–1,100 cm^–1^, as well as the bending mode of Si–O–Si at 470 cm^–1^, is difficult due to their relative weakness.

### 3.3 Wettability

The hydrophilicity degree of biomaterials represents a crucial parameter in determining their interaction with cells. It is well documented that hydrophobic surfaces with low wettability hinder cellular adhesion and compromise material integration with the surrounding tissue. Conversely, hydrophilic fibers better mimic the natural biological environment, enhancing cellular adhesion and proliferation.^55^

The wettability of electrospun blend mats was determined by contact angle assessment and modulated by the PPSG concentration. Fibers containing only PCL exhibited hydrophobic characteristics with a contact angle of 119 ± 8°. Enzymatically synthesized glycerol-based polyesters such as PGS, PPSG, and PGO exhibit high hydrophilicity due to hydroxyl groups linked to their structure.^56–58^ Therefore, mixed fibers containing PPSG (Figure 4) showed hydrophilic behavior as the PPSG concentration increased (28 ± 3° for PCL/5PPSG and 40 ± 5° for PCL/10PPSG). The contact angle for the PCL/20PPSG sample was not detectable, as the water droplet rapidly penetrated and spread on the sample surface.

**Figure 4:**
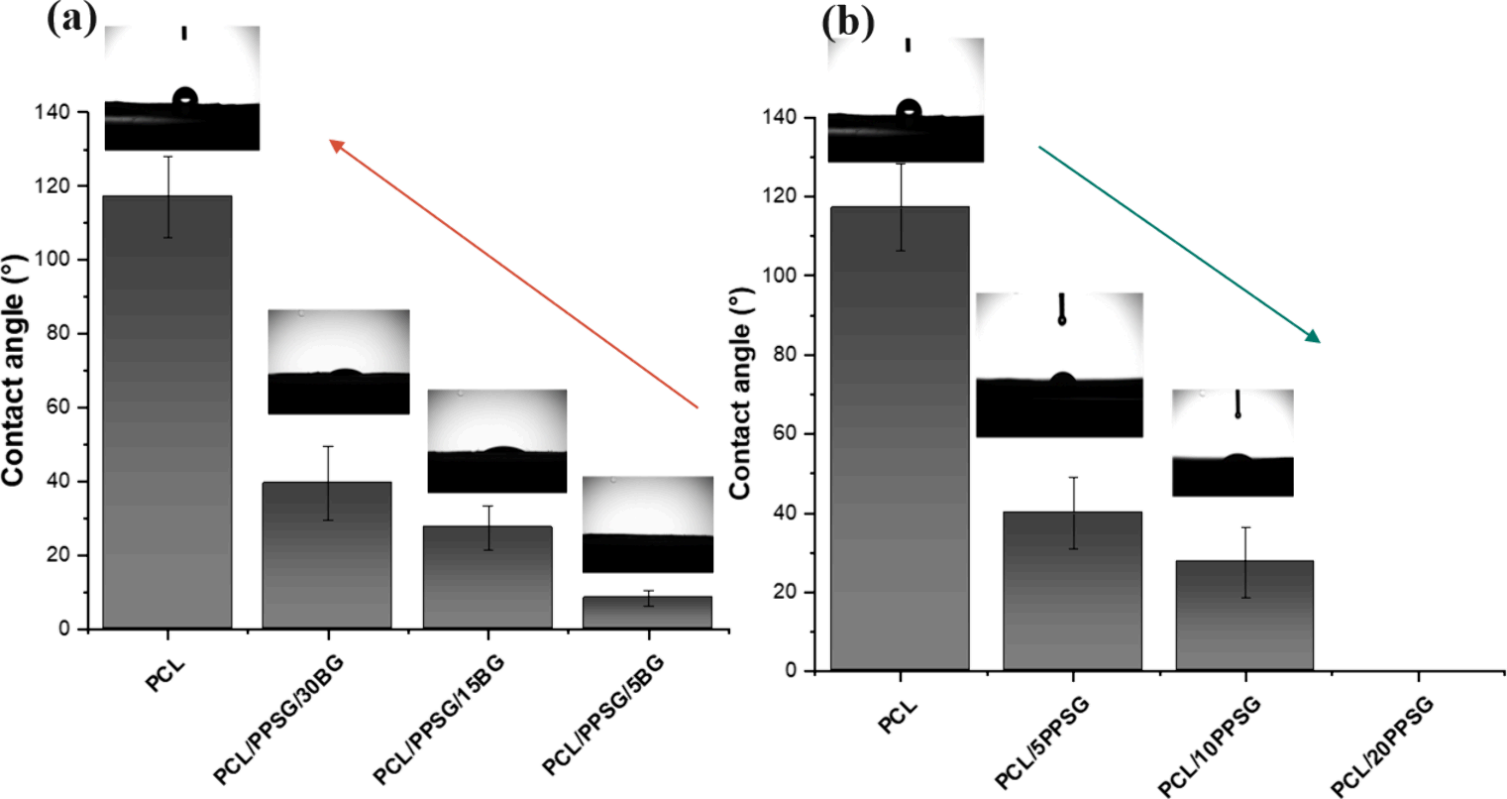
Degree of wettability of electrospun mats, as determined by contact angle measurement. (**a**) Composite mats containing PCL/PPSG blends and BG 45S5 bioactive glass. (**b**) Electrospun PCL mats with varying PPSG fractions.

Conversely, it was observed that fiber mats containing bioactive glass particles exhibited less hydrophilic behavior as the BG concentration increased, with contact angles of 28 ± 4° and 40 ± 5° for samples PCL/10PPSG/15BG and PCL/10PPSG/30BG, respectively. Literature reports indicate that electrospun PCL mats exhibit contact angles in the range of 133°-98°,^59–61^ and blends of PCL with PGS (1:0.5) have no measurable contact angles.^10,11,62^ The literature has documented that extreme hydrophobic or hydrophilic properties are unfavorable for promoting cellular adhesion. The ideal wettability for adequate cellular attachment is 40° to 70°.^63^ Therefore, the analysis shows that PPSG and BG strongly affect the wettability of PCL/10PPSG/BG blends, and their concentration would be vital to controlling hydrophilicity (Figure 4).

### 3.4 Degradation in PBS Solution

When introduced into biological environments, degradable biomaterials undergo surface or bulk degradation, exhibiting progressive alterations in their physicochemical properties over time. In the case of aliphatic polymers like PCL and PPSG, hydrolytic degradation of the ester bonds occurs at varying rates. Typically, glycerol-based polyesters such as PPSG are fully degraded within a few weeks due to their inherent hydrophilicity.^64,65^ Meanwhile, PCL demonstrates a slower degradation rate due to its semicrystalline nature and molecular weight.^66^ From this perspective, PCL/PPSG blends emerge as promising candidates for developing hybrid materials with controlled degradation kinetics. In vitro immersion tests in PBS solution were conducted to explore degradation and weight loss was monitored over 35 days for fibers comprising pure PCL, PCL/PPSG, and PCL/PPSG/BG blends, as shown in Figure 5.

**Figure 5:**
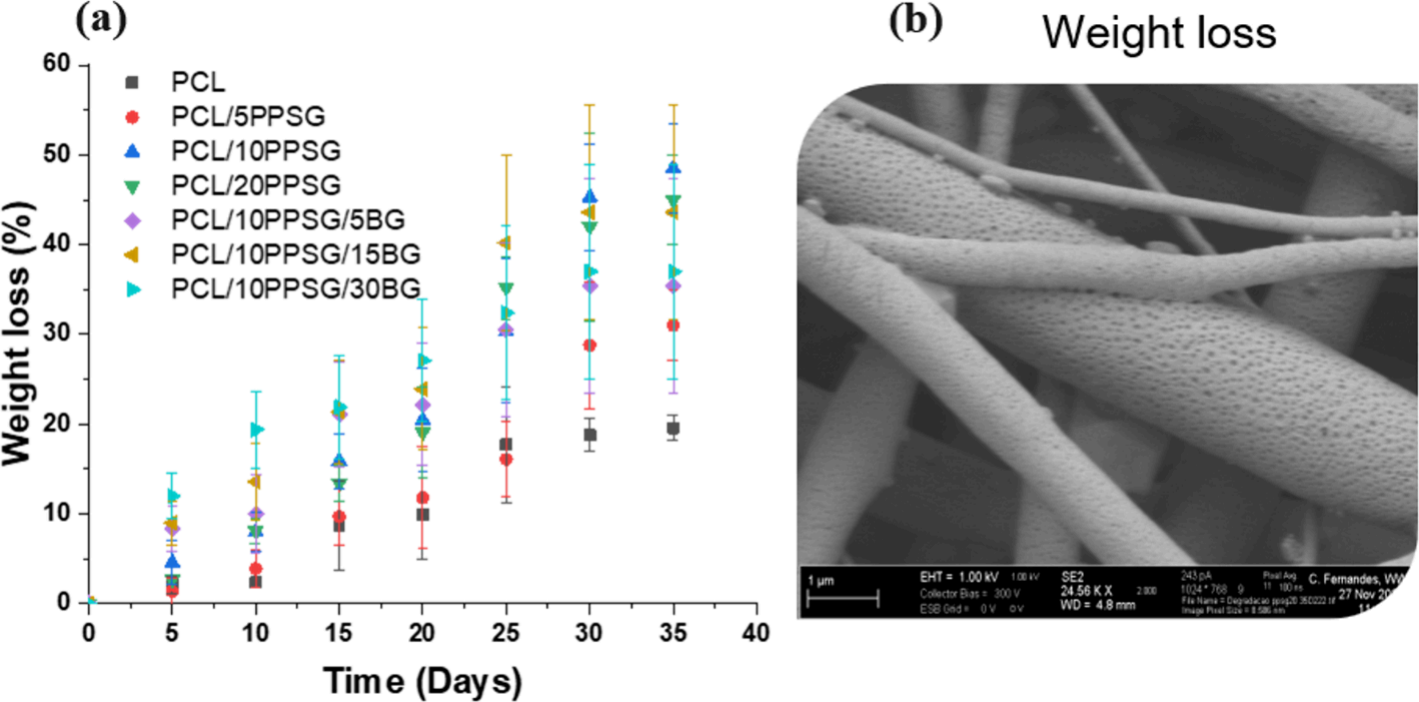
Weight loss during submersion in PBS for 35 days (**a**) and SEM micrograph of samples after 35 days of immersion PCL/10PPSG fiber (**b**).

The weight loss dynamic profiles, depicted in Figure 5a, revealed rapid degradation of PCL/PPSG fiber mats attributed to hydrolytic cleavage of ester bonds in both PCL and PPSG components. Microscopic analysis depicted significant degradation of PCL/20PPSG samples (Figure 5b), evidenced by pore and crack formation likely due to the leaching of PPSG degradation products. In contrast, pure PCL samples exhibited a lower weight loss. Moreover, by the end of 35 days in PBS, the pH of the pure PCL samples hardly changed, while the pH of the PBS degradation medium showed an acidification trend as PPSG concentrations increased by 5%, 10%, and 20%, reaching values of 7.3, 7.0, and 6.9, respectively. Prior studies have also noted common leaching of unreacted glycolic polyesters, such as PGS, in electrospun fibers.^66^

Incorporating BG particles into PCL/PPSG mats led to small pore formation, indicating bioactive glass particle leaching (blue arrow, Figure 6l). Furthermore, weight loss measurements confirmed accelerated degradation of PCL/PPSG blends compared to pure PCL mats, with higher PPSG concentrations showing more significant weight loss after 35 days in PBS incubation. Overall, PCL/PPSG blends exhibited a weight loss of approximately 45% ± 5 after 35 days, highlighting the higher degradation rates than PCL/PPSG mats (12%).^18,67^ Therefore, the measured results of degradation behavior underscore the significance of understanding polymer interactions and blend compositions for tailored degradation kinetics in biomedical applications.

**Figure 6:**
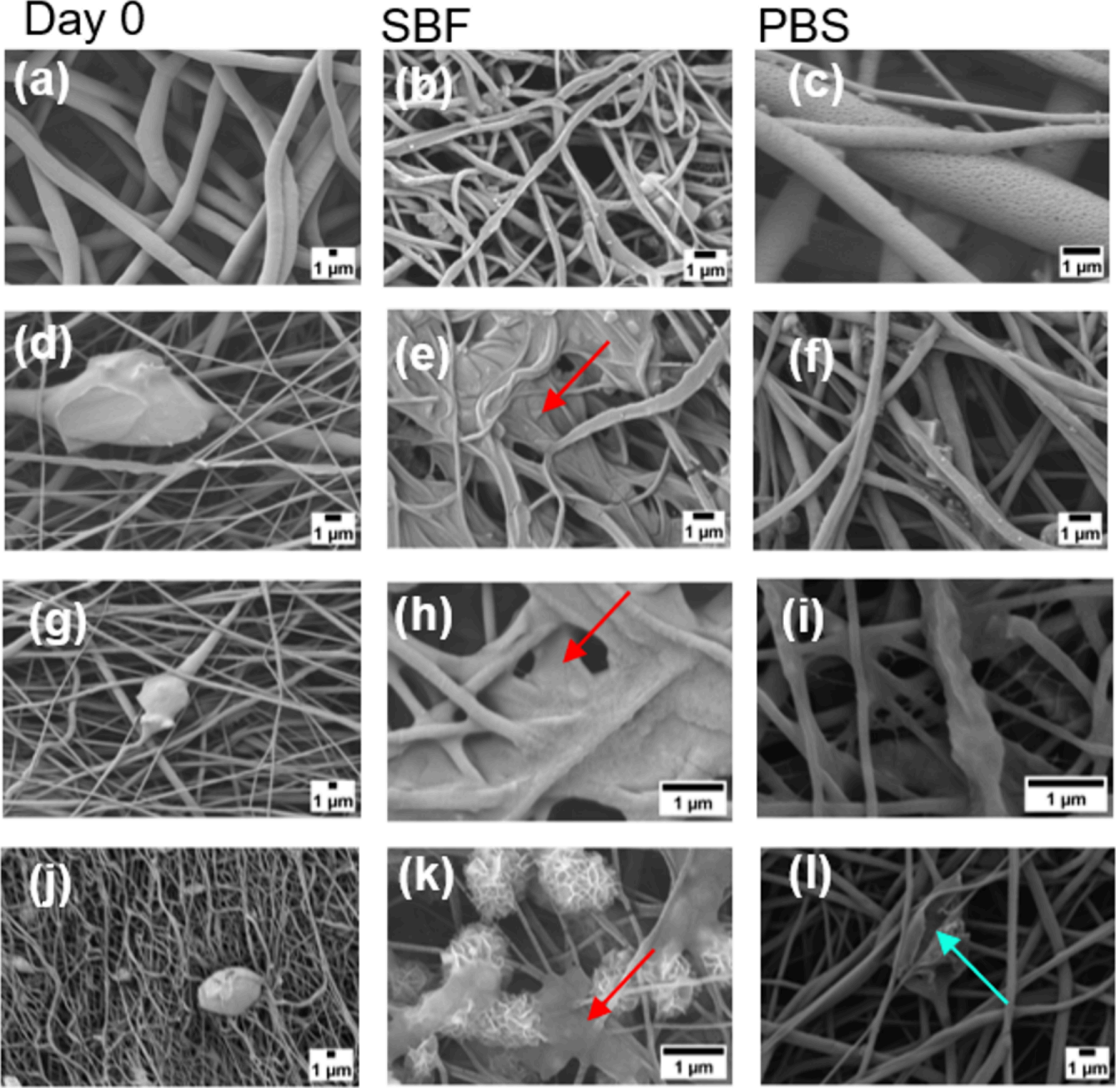
SEM images of fibers containing BG particles after inoculation in SBF and PBS for 14 days: (**a**,**b,c**) PCL/10PPSG; (**d,e,f**) PCL/10PPSG/5BG; (**g,h, i**) PCL/10PPSG/15BG; (**j,k,l**) PCL/10PPSG/30BG. The red arrow indicates Hydroxyapatite.

### 3.5 *In Vitro* Acellular Bioactivity

The ability to form hydroxyapatite on the mat surfaces was evaluated as an indicator of fiber bioactivity.^38^ Fibers without the presence of bioactive glass were also evaluated as control samples, and no evidence of hydroxyapatite was found after 14 days of analysis (Figure 6b). The other representative SEM micrographs of fibers containing BG particles after 14 days in SBF and PBS solutions are shown in Figure 6.

Hydroxyapatite deposition was observed in the composite fibers with the addition of BG 45S5 (Figure 6d). Samples PCL/PPSG/5BG, PCL/PPSG/15BG, and PCL/PPSG/30BG showed biomineralization effects (Figures 6a and 6b). The morphology of the deposited layer exhibited variations observed in the PCL/10PPSG/30BG sample (Figure 6k) at the end of immersion in SBF solution, compared to the samples PCL/PPSG/5BG and PCL/10PPSG/15BG in SBF (Figure 6e and 6h), exhibiting the characteristic morphology of hydroxyapatite, which has been extensively documented in the literature.^25,68,69^ As no morphological change was noted for those samples when immersed in buffered PBS saline solution, it can be concluded that the change is related to the BG concentration, which caused the mineralization of the samples, as frequently reported in the literature for similar BG containing fibers.^34^

The evaluation and investigation of the formation of hydroxyapatite on the fibers was carried out through observations of FTIR spectra, which are presented in Figure 7. Peaks around 560 and 600 cm^–1^ are related to the phosphate group and were detected in all samples after 3 days of immersion in SBF solution, as reported in Table S2. Furthermore, additional peaks attributed to the stretching vibration of silica (Si–OH) were observed at 1050 cm^–1^ in the spectra of all fibers immersed in the SBF solution.^70^ Moreover, a wide peak at 3402 cm^–1^ and the ester bonds at 1721 cm^–1^ qualitatively decreased in intensity after immersion in SBF solution. This behavior suggests that OH (hydroxyl) and COOH– (carboxyl) groups are present in the PCL/PPSG mats. It is important to note that these changes indicate functional group presence but they do not provide quantitative information about their concentration. Usually, PCL/PPSG undergoes hydrolysis through ester bonds, transforming them into oligomers with hydroxyl and carboxylic terminal groups. Considering SEM micrographs, FTIR characterization, and weight loss analysis in PBS medium, leaching or degradation of PGS from PCL/PPSG mixtures in the medium is plausible. Additionally, the samples present peaks related to stretching vibrations at 1030, 810, and 460 cm^–1^, attributed to Si–O–Si, bonds, indicating the presence of the silica network in the BG particles.^71^ Previous work also reported the presence of vibration bands in this region due to phosphate groups with stretching vibration, indicating hydroxyapatite growth on the material’s surface.^71^

**Figure 7:**
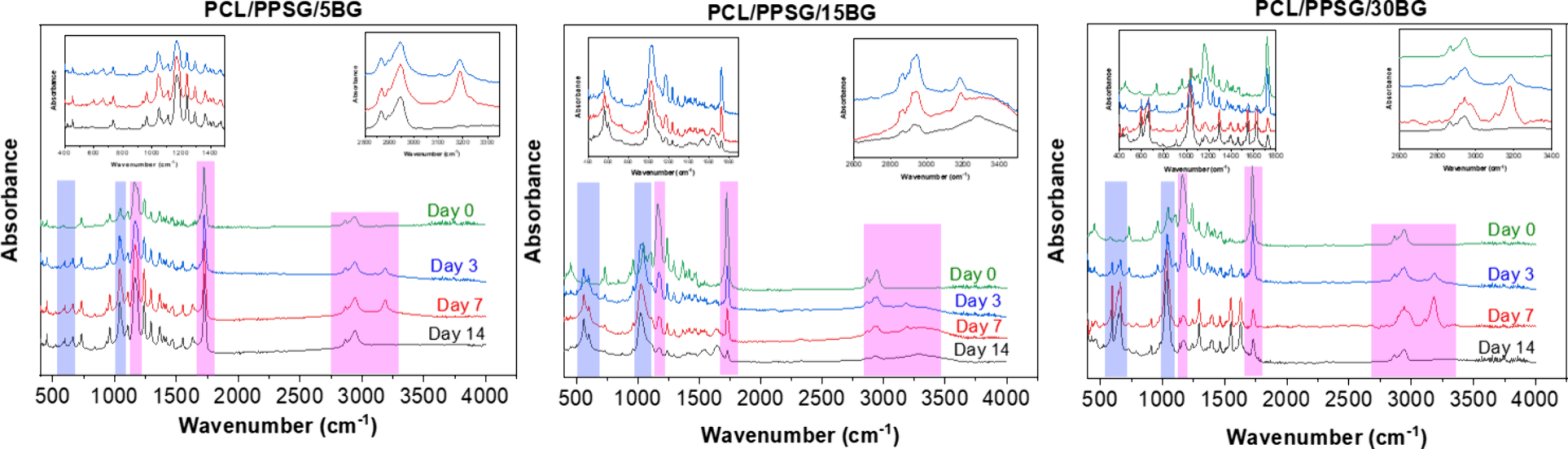
FTIR spectra of samples immersed in SBF over 14 days.

XRD analysis confirmed the presence of hydroxyapatite in the fibers containing BG, as illustrated in Figure 8. All samples presented characteristic PCL peaks and evidence of bioactivity was demonstrated after the immersion period. Specifically, the peak at 2θ = 32° and 46,7° can be attributed to the presence of hydroxyapatite.^71–73^ This result is significant for tissue engineering applications, as it confirms for the first time the bioactivity of BG 45S5 particles embedded in the new biopolymer matrix.

**Figure 8:**
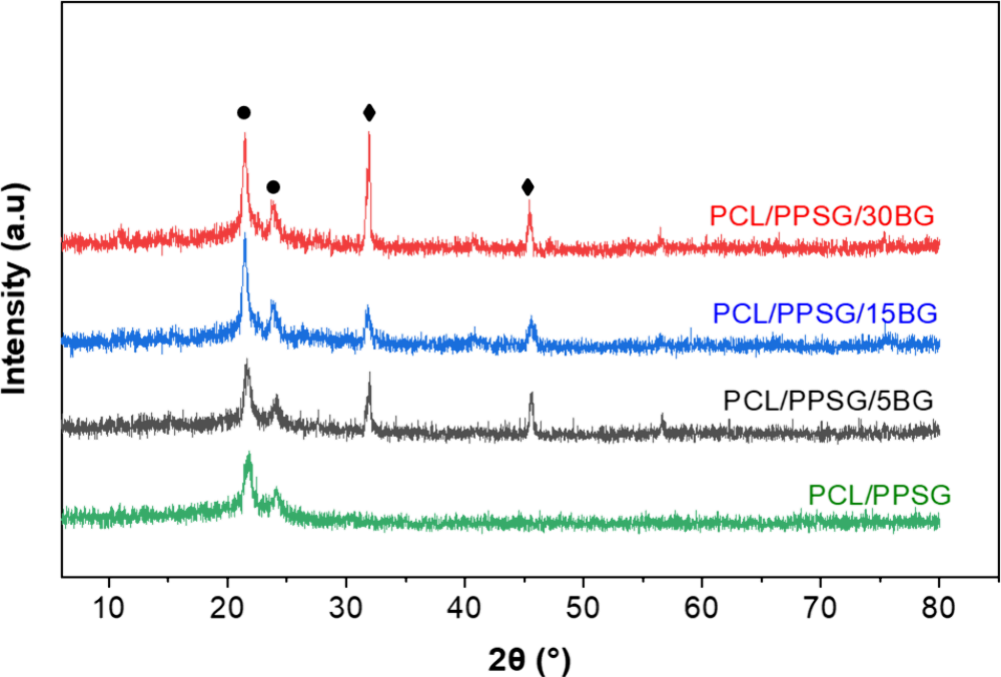
XRD patterns of samples immersed in SBF after 14 days. The presence of hydroxyapatite (⧫) is marked by the peaks at 2θ = 32° and 46.7°. The crystallinity of PCL (●) is marked by the peaks at 2θ = 21.4° and 23.7°.

### 3.6 Mechanical Properties

Understanding the mechanical properties of biomaterials is important for developing useful materials for tissue engineering. The mechanical properties of PCL/PPSG fibers and composite fibers incorporating bioactive glasses (PCL/PPSG/BG) was investigated by tensile testing, focusing on the ultimate tensile strength (σ) and Young’s modulus, E (Figure 9 and Table 1).

**Figure 9:**
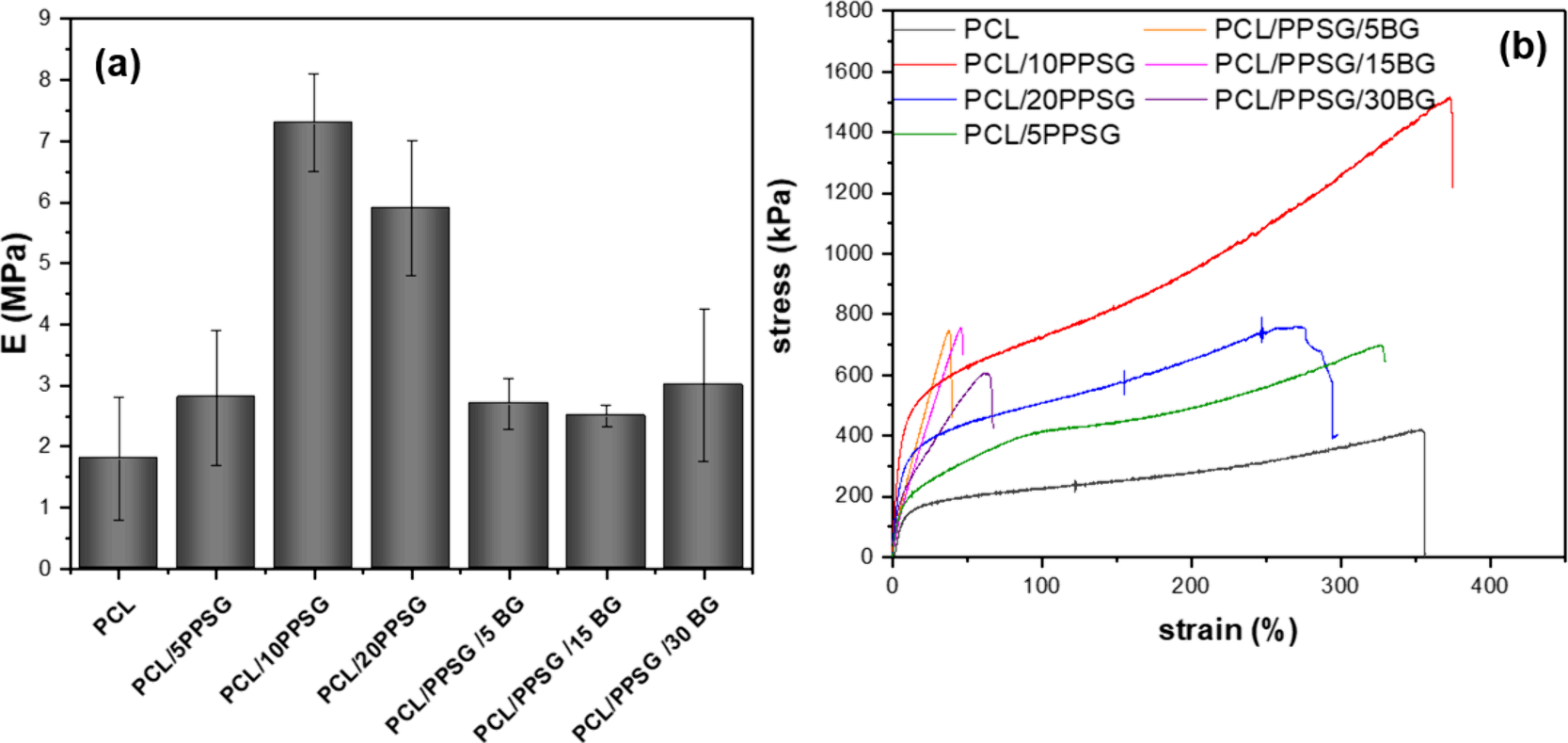
Results of (**a**) Young’s Modulus, E measurements and (**b**) stress–strain curves of PCL/PSG fibers and fibers incorporating bioactive glass (PCL/PPSG/BG).

**Table 1:** Correlation of Tensile Test Results with Fiber Diameter

Sample	Fiber Diameter (μm)	E (MPa)	σ (MPa)	Elongation at Break (%)
**PCL**	1.8 ± 0.2	1.7 ± 1	0.4 ± 0.1	355 ± 45
**PCL/5PPSG**	2.2 ± 0.4	2.1 ± 1.1	0.7 ± ± 0.1	330 ± 27
**PCL/10PPSG**	2.2 ± 0.2	6.0 ± 0.8	1.5 ± ± 0.1	374 ± 33
**PCL/20PPSG**	1.7 ± 1.2	8.8 ± 1.5	0.7 ± ± 0.1	295 ± 25
**PCL/10PPSG/5BG**	0.93 ± 0.4	2.7 ± 0.3	0.7 ± ± 0.1	43 ± 4
**PCL/10PPSG/15BG**	0.55 ± 0.3	2.5 ± 0.1	0.8 ± 0.1	47 ± 7
**PCL/10PPSG/30BG**	0.63 ± 0.3	3 ± 0.3	0.6 ± 0.3	69 ± 3

One can observe that the addition of PPSG to the PCL blend resulted in a significant increase in Young’s modulus, from 2 ± 0.8 MPa to 3 ± 1 MPa, 7 ± 1 MPa, 6 ± 0.8 MPa, as the PPSG concentration increased (PCL, PCL/5PPSG; PCL/10PPSG; PCL/20PPSG, respectively). This increase indicates greater stiffness of the material, attributed to the improved mechanical properties conferred by the presence of PPSG. Similar behavior was reported in the literature where the addition of PGS to PCL increased the stiffness of the fibers.^40^ These results may be related to the interaction between PPSG and PCL creating a more cohesive polymer blend with a larger average fiber diameter and, therefore, with greater stress transfer between the chains in relation to pure PCL. Furthermore, we observed a notable increase in the σ of the PCL and PPSG blend compared to pure PCL fibers. While PCL fibers presented a σ of 0.4 MPa, the PCL/PPSG blends achieved a σ of 1.5 MPa. This increase suggests a higher ability of the material to withstand maximum tensile loads before failure. On the other hand, although the σ analysis showed that PPSG improved the tensile strength of PCL/PPSG blends, the failure strain and Young’s modulus values decreased with the addition of bioactive glasses (PCL/PPSG/BG). These results may be connected to the distribution of fiber morphology and BG particles in the polymeric solution, which causes weak interface points between the BG particles and the polymer. Similar results have been reported in the literature for particle addition in PCL and Chitosan fibers. The reduction in Young’s modulus and σ values occurs due to the presence of inorganic particles in high concentration (30 wt %).^34,40^ Thus, adding BG particles aims to introduce relevant tools for tissue regeneration. However, particle addition also affects the ultimate mechanical behavior of the fibers.

A key factor affecting the mechanical stability of PCL/PPSG/BG scaffolds is the absence of strong covalent interactions between the BG particles and the polymer matrix. Unlike chemically bonded systems, where molecular interactions enhance load transfer, the BG-polymer interface in this study relies on weak physical interactions such as hydrogen bonding and van der Waals forces. This weak interfacial adhesion can result in localized stress accumulation, leading to early mechanical failure under load. As a consequence, the presence of BG, despite its bioactivity benefits, introduces potential points of structural weakness.

Furthermore, the distribution of BG particles within the polymer matrix plays a crucial role in determining scaffold performance. As observed in Figure 2 (SEM/EDX analysis), mapping of the BG dispersion reveals regions of particle aggregation, which likely act as stress concentration points, reducing the overall mechanical strength. These clustered regions may disrupt the homogeneous transfer of load through the scaffold, increasing susceptibility to the initiation and propagation of mechanical stress points. A more uniform dispersion of BG particles would favor stress dissipation, improving mechanical integrity.

Moving forward, investigating the effects of controlled reduction of BG particle size and optimizing BG concentration within the polymer matrix should be the focus of future studies. This would allow for a better balance between increased bioactivity and mechanical stability, ensuring that the scaffold maintains its structural integrity while still promoting cellular interaction and mineralization.

The analysis shows that the blends presented higher mechanical properties compared to some soft tissues for example. Young’s modulus for myocardial tissue is expected to be in the range of 0.02 to 0.5 MPa, while its tensile strength is in the range 3–15KPa.^74^ However, it is important to consider that higher values of the mechanical proprieties of biomaterials should not harm their necessarily final performance. Theoretical simulations have demonstrated that a stiffer cardiac patch can positively contribute to the mechanical properties of the myocardium, which reduces stress on the cardiovascular wall, an essential factor in the propensity for infarctions.^74,75^ This behavior suggests that the PCL/PPSG blend is particularly promising for applications where mechanical strength is crucial.

### 3.7 Cell Viability

Cell viability was investigated through the proliferation of NHDF cells on electrospun mats of PCL/PPSG, as well as on fibers containing bioactive glass particles PCL/PPSG/BGs. It was found that NHDF cells proliferated and spread well on all fibers over the 3-day culture period. Cell metabolic activity increased considerably during this period, as evidenced by the results it is shower in Figure 10. Initially, a lower number of cells was observed on the PCL and PCL/PPSG samples in the first 24 h of cell growth, possibly due to the rapid degradation of electrospun surfaces, which may reduce cell adhesion capacity due to medium acidification, as reported in the literature.^49^

**Figure 10:**
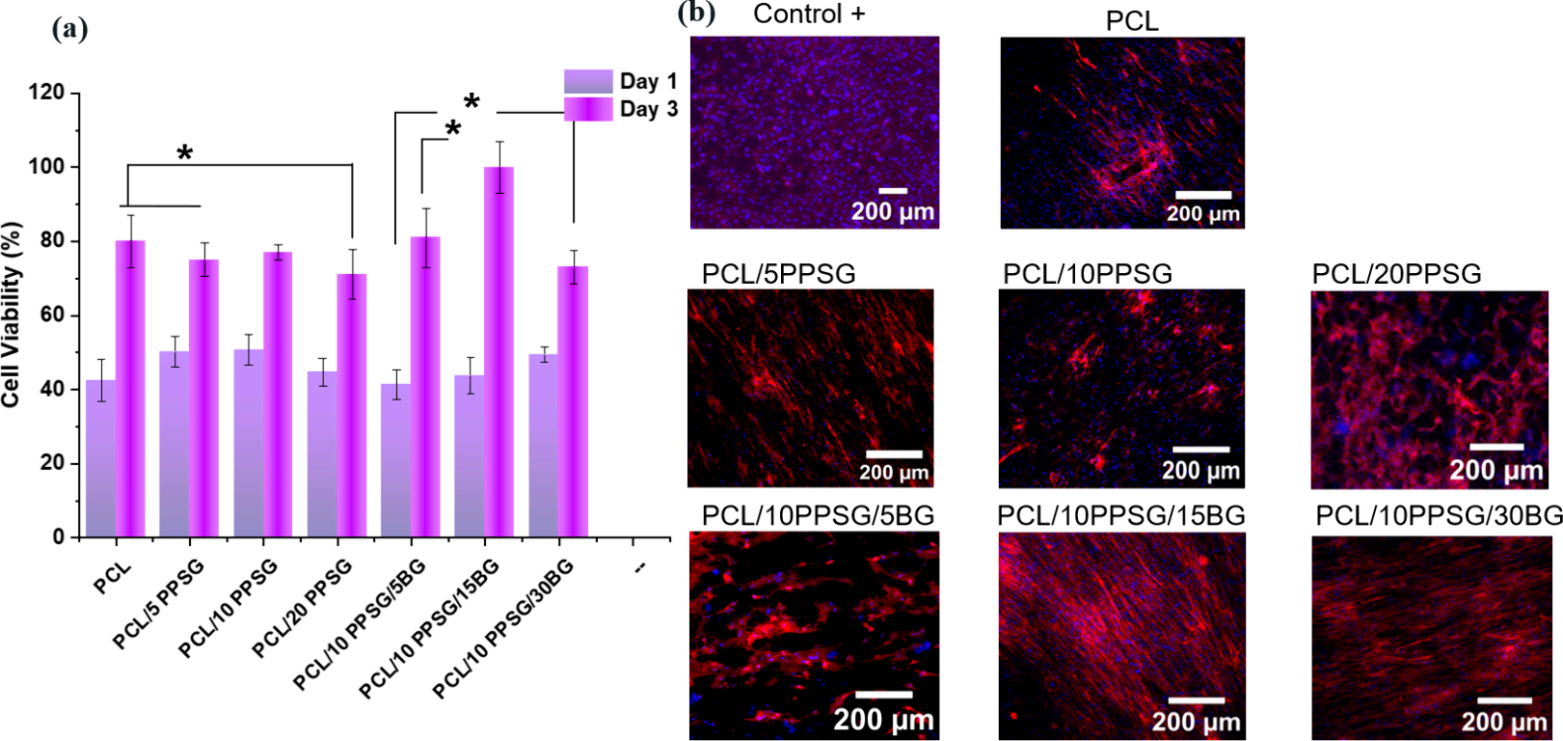
(**a**) Cell viability was analyzed using a cellular dehydrogenase staining assay at a wavelength of 450 nm (*p < 0.05). (**b**) Fibroblasts cultured on fiber blends were analyzed using fluorescence microscopy, with DAPI staining in blue (cell nuclei) and rhodamine-phalloidin in red (actin filaments).

However, this effect was mitigated with the addition of BG particles, indicating that ion release from BG stimulates cell growth. Analysis of the samples after 3 days of cell growth revealed an increase in cell viability, especially in samples containing 15% BG particles in the composition. This result is consistent with previous studies that highlighted the favorable effect of BGs on cell viability, attributing it to ion release that stimulates mRNA-level processes, improving cell adhesion and proliferation.^12,76^ Morphology and differences in mechanical properties of the fibers also play an important role in the biological response. According to Chen et al., a decrease in fiber diameter is associated with increased cell adhesion due to increased specific surface area and consequent adsorption of proteins, which is crucial for adhesion on fiber surfaces.^77,78^ Analogous to these observations, it was found that the fiber diameter of PCL/PPSG was greater than that of PCL/PPSG/BG, affecting cell adhesion.

The sudden change in the mechanical properties of the fibers can also be a factor influencing cell adhesion. Generally, greater mechanical strength can provide stability for cell development, while more deformable, less rigid scaffolds tend to present lower cell proliferation rates.^79^ In the case of composites with BG, a decrease in mechanical elasticity can favor proliferation over 3 days. Indeed, PCL/PPSG/BG composite fibers showed increased cell viability at the end of the analysis time. Another critical factor is that during this period, ion release from BG occurred, likely overcoming the polymer’s physical barrier and assisting cell attachment and proliferation.

Furthermore, analysis of Figure 10b, which shows the nuclei and cell filaments of stained NHDF cells cultured on fibers for 3 days, revealed that the cells spread and interconnected across the surface of the samples. Notably, the red staining indicated that there was elongation of the cell filaments in all samples. This elongation indicates a well connected network, representing effective cell adhesion and proliferation. These results corroborate the WST-8 analysis, in which the PCL/PPSG/15BG sample exhibited the highest cell viability and, consequently, the greatest stretching for interconnection of the filaments. Furthermore, the analysis also agrees with previous investigations in which polymer blends contaning glycerol have superior cell adhesion and proliferation.^51,80^

## 4 Conclusions

Author: Homogeneous and bead-free fiber mats were obtained from PCL/PPSG blends. Bioactive glass 45S5 particles were integrated and homogeneously dispersed into the electrospun fibers. Remarkably, the copolymer PPSG improved the mechanical properties of the fibers, while incorporating BG particles created stress points that reduced the mechanical properties. The presence of PPSG significantly contributed to the hydrophilicity and rapid biodegradability of the fibers, even at low concentrations. Cell viability studies showed that the presence of BG particles contributed to cell attachment and growth. Furthermore, the 30% BG concentration led to hydroxyapatite formation upon immersion in SBF, revealing that BG concentration is the key factor for the modulation of mineralization. While PCL was used as a reference for cell viability measurements, a direct comparison with collagen-based scaffolds or other biomaterial controls was not included in this study. Future studies should explore additional biological benchmarks to further validate the biocompatibility and functional performance of these scaffolds in a physiologically relevant context. The results of this work are promising and can serve as a foundation for upcoming comparative studies with similar biopolymer/BG fibrous scaffolds for possible application in soft tissue engineering.

## Supplementary Material



## Notes

DSC thermograms and summary table of thermal properties (T_m_, Tc, ΔH) for electrospun PCL and PCL/PPSG fibers; table of FTIR characteristic absorption bands for composite mats; EDX spectrum of electrospun PCL/PPSG/BG fibers (PDF)
