# Supplementary material for: Incorporation of Poly(propylene succinate-co-glycerol succinate) (PPSG) as a Renewable Additive in Electrospun PCL Fibers with Bioactive Glass Particles for Soft Tissue Engineering
Source: ACS Appl Bio Mater. 2025 Jun 3;8(6):4791–804. doi: 10.1021/acsabm.5c00176 (PMC12175137; doi:10.1021/acsabm.5c00176)
Supplement: Supplementary file 1 [file mt5c00176_si_001.pdf]

Supporting Information.

Title: Incorporation of Poly (propylene succinate-co-glycerol succinate) PPSG as a Renewable Additive in Electrospun PCL Fibers with Bioactive Glass Particles for Soft Tissue Engineering

Clara Dourado Fernandes[a,b] *, Alina Grünewald [b]; Zoya Hadzhieva[b], Bruno Francisco Oechsler [a], Claudia Sayer [a], Pedro H. Hermes de Araújo [a] *, Aldo R. Boccaccini[b]*

[a] Department of Chemical Engineering and Food Engineering, Federal University of Santa Catarina, 88040-900, Florianópolis, SC, Brazil.

[b] Institute of Biomaterials, Department of Materials Science and Engineering, University of Erlangen-Nuremberg, Cauerstr. 6, 91058 Erlangen, Germany

*Corresponding author

clara.dourado.fernandes@fau.de
pedro.h.araujo@ufsc.br
aldo.boccaccini@fau.de

Table S1. Thermal properties of electrospun PCL and PCL/PPSG fibers determined by DSC analysis. The table presents the melting temperature (T_m), crystallization temperature (T_c), and enthalpy of fusion (ΔH) for electrospun fiber samples of pure PCL and PCL/PPSG blends (5%, 10%, and 20% PPSG).

**Sample**	Tm (°C)	Δ*H*(J/g)	Tc (°C)
**PCL**	53.1	3.8	33.8
**PCL/5PPSG**	51.9	3.8	33.3
**PCL/10PPSG**	52.3	3.8	32.9
**PCL/20PPSG**	52.3	3.1	32.9

Melting temperature (T_m), crystallization temperature (T_c), enthalpy of fusion (ΔH).

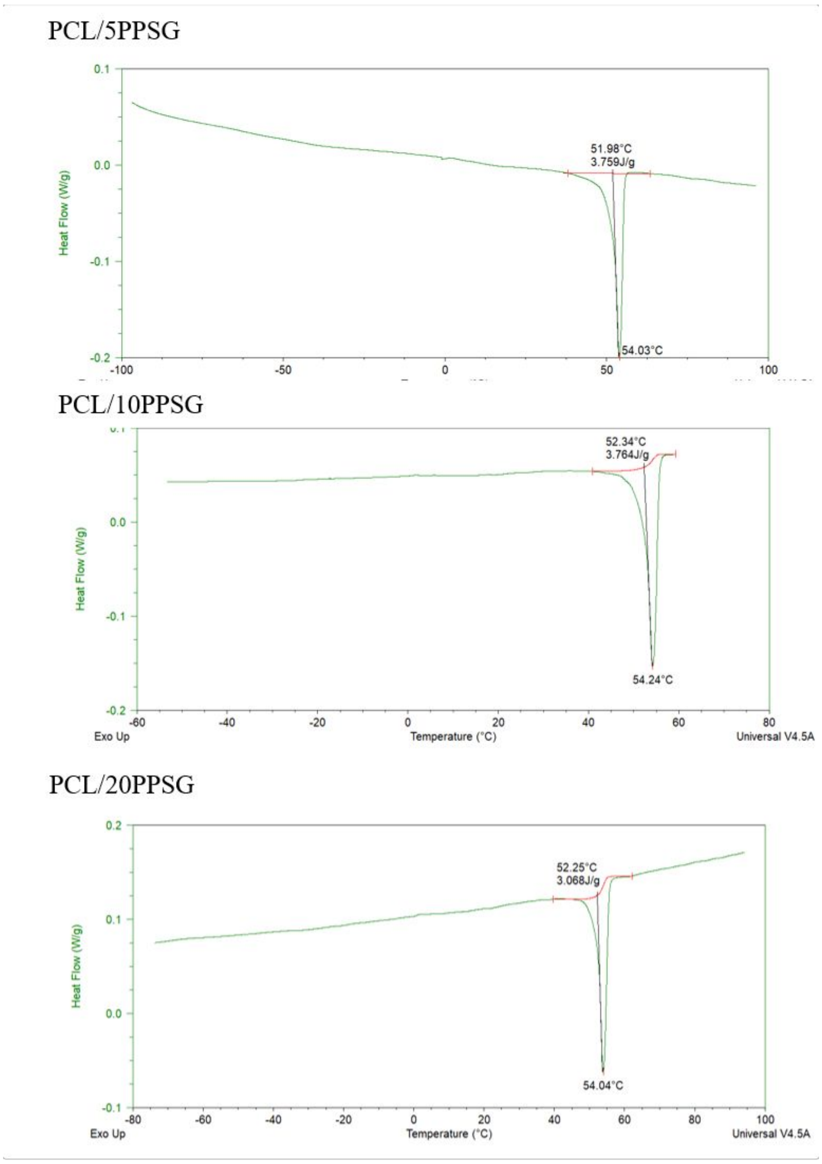

Figure S1. DSC thermograms of electrospun fibers of PCL and PCL/PPSG blends.

Table S2. Characteristic FTIR absorption bands of PCL electrospun mats with the "green" PPSG and BG bioactive glass and their designation according to [15,44].

**Absorption band (cm^{-1})**	**Description**
2935; 2864; 1365	Stretching of alkyl group (-CH2)
1726	Stretching of carbonyl group (C=O)
1296	Backbone C-C and C-O stretching
1166	Stretching of ester group (C-O)
900; 1100	Stretching of silicate group (Si-O-Si and Si-O)
470	Bending of silicate (Si–O–Si)

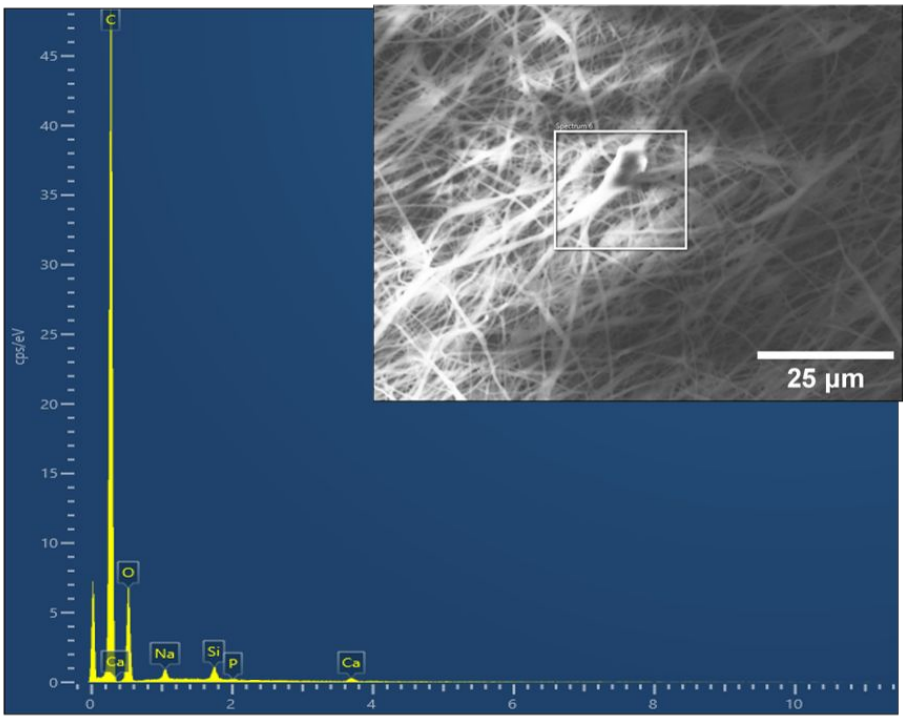

Figure S2. EDX analysis of electrospun fiber with PCL/PPSG/5BG bioglass
